# *Pseudmonas cannabina* pv. *alisalensis* TrpA Is Required for Virulence in Multiple Host Plants

**DOI:** 10.3389/fmicb.2021.659734

**Published:** 2021-04-20

**Authors:** Nanami Sakata, Takako Ishiga, Yasuhiro Ishiga

**Affiliations:** Faculty of Life and Environmental Sciences, University of Tsukuba, Tsukuba, Japan

**Keywords:** *Pseudomonas cannabina* pv. *alisalensis*, tryptophan, type three secretion system, coronatine, cabbage, oat

## Abstract

*Pseudomonas cannabina* pv. *alisalensis* (*Pcal*) causes bacterial leaf spot and blight of Brassicaceae and Poaceae. We previously identified several potential *Pcal* virulence factors with transposon mutagenesis. Among these a *trpA* mutant disrupted the tryptophan synthase alpha chain, and had an effect on disease symptom development and bacterial multiplication. To assess the importance of TrpA in *Pcal* virulence, we characterized the *trpA* mutant based on inoculation test and *Pcal* gene expression profiles. The *trpA* mutant showed reduced virulence when dip- and syringe-inoculated on cabbage and oat. Moreover, epiphytic bacterial populations of the *trpA* mutant were also reduced compared to the wild-type (WT). These results suggest that TrpA contributes to bacterial multiplication on the leaf surface and in the apoplast, and disease development. Additionally, several Brassicaceae (including Japanese radish, broccoli, and Chinese cabbage) also exhibited reduced symptom development when inoculated with the *trpA* mutant. Moreover, *trpA* disruption led to downregulation of bacterial virulence genes, including type three effectors (T3Es) and the phytotoxin coronatine (COR), and to upregulation of tryptophan biosynthesis genes. These results indicate that a trade-off between virulence factor production and *Pcal* multiplication with tryptophan might be regulated in the infection processes.

## Introduction

The foliar plant bacterial pathogen *Pseudomonas syringae* causes economically important diseases in a wide range of plants (Agrios, 2005). *P. syringae* colonizes leaf surfaces (epiphytic) of host plants, enters natural opening sites, including stomata, and then multiplies in the leaf interior (apoplast) (Xin and He, 2013). During infection processes, *P. syringae* suppresses plant basal defense by using virulence factors, such as specialized protein secretion systems, toxins, plant hormones, bacterial surface attachment factors, flagella, and siderophores (Xin and He, 2013). *P. syringae* pv. *tomato* (*Pst*) DC3000 also infects *Arabidopsis* (Whalen et al., 1991), and is used as a model pathogen to study plant-bacterial interactions. *Pst* DC3000 is a highly aggressive pathogen once inside host tissue, and uses many type three effectors (T3Es) and the phytotoxin coronatine (COR; Buell et al., 2003; Feil et al., 2005). The function of these two *Pst* DC3000 virulence factors have been well characterized at the molecular level. However, Boch et al. (2002) demonstrated that a wide range of plant-induced loci in *Pst* DC3000 included not only virulence associated genes such as *hrp* genes and COR biosynthesis genes, but also genes involved in stress tolerance, polysaccharide synthesis, nutrient uptake, amino acid assimilation, and carbon metabolism. Although virulence associated genes related to type three secretion system (T3SS) and COR have been investigated well in the *P. syringae* virulence, functions of other genes including amino acid metabolism remain largely unclear.

*Pseudomonas cannabina* pv. *alisalensis* (*Pcal*) causes bacterial leaf spot and blight of Brassicaceae and Poaceae (Takikawa and Takahashi, 2014). *Pcal* was formally classified as *P. syringae* pv. *maculicola* (*Psm*). Although *Pcal* and *Psm* have similar characteristics, these two pathogens are defined by some bacteriological characteristics, genetic traits, and their ability to infect monocot plants such as oat (*Avena sativa*) and timothy (*Phleum pretense*) (Cintas et al., 2002; Bull et al., 2010; Takikawa and Takahashi, 2014). Our recent study identified several potential *Pcal* virulence factors, including T3SS, membrane transporters, transcriptional factors, and amino acid metabolism (Sakata et al., 2019). Among these, the mutants which were disrupted in amino acid metabolism showed no pathogenicity, similar to a T3SS mutant (Sakata et al., 2019). A *trpA* (encoding tryptophan synthase alpha chain) mutant exhibited reduced disease symptom development and bacterial multiplication (Sakata et al., 2019). Helmann et al. (2019) conducted a genome wide screening to identify *P. syringae* pv. *syringae* (*Pss*) B728a virulence factors, and identified that *trpA* contributes to bacterial fitness on both the leaf surface and in the apoplast. Tryptophan is the least abundant amino acid in leaf exudates from many plant species (Morgan and Tukey, 1964; Rico and Preston, 2008). Although amino acid metabolism is essential for bacterial growth, the function of amino acid metabolism in plant bacterial virulence has not been investigated.

Here, we showed that TrpA contributes to *Pcal* virulence in successful infection processes. Moreover, *trpA* mutant expression profiles analysis showed downregulation of virulence related genes, including T3Es and COR, and upregulation of tryptophan biosynthesis related genes compared to WT. These results suggest that trade-off between virulence factor production and tryptophan biosynthesis might be present in bacterial infection processes.

## Materials and Methods

### Bacterial Strains, Plasmids, and Growth Conditions

The bacterial strains and plasmids used in this study are described in Supplementary Table 1. *P. cannabina* pv. *alisalensis* strain KB211 (*Pcal* KB211) was used as the pathogenic strain to inoculate cabbage, oat, Japanese radish, broccoli, and Chinese cabbage. *Pcal* wild-type (WT) was grown on King’s B (KB; King et al., 1954) medium at 28°C. NB35, NF2, NF34, NI13, NH11, NM37, and NN31 were grown on KB containing kanamycin (10 μg/ml) (Km). *trpA* mutant complemented with pDSK-*trpA* was grown on KB containing Km (10 μg/ml) and gentamicin (Gen) (25 μg/ml) (Supplementary Table 1). Before *Pcal* inoculation, bacteria were suspended in sterile distilled H_2_O, and the bacterial cell densities at 600 nm (OD_600_) were measured using a Biowave CO8000 Cell Density Meter (Funakoshi, Tokyo, Japan).

### Bacterial *in vitro* Growth Measurements

Wild-type, the *trpA* mutant, and the *trpA* mutant complemented with pDSK-*trpA* were grown at 28°C on Luria-Bertani (LB; Sambrook et al., 1989) medium. The bacterial suspensions were standardized to an OD_600_ of 0.01 with LB, and bacterial growth was measured at OD_600_ for 24 h. WT and the *trpA* mutant were also grown at 28°C in mannitol-glutamate (MG; Keane et al., 1970) medium. The bacterial suspensions were standardized to an OD_600_ of 0.1. L-tryptophan (FUJIFILM Wako Pure Chemical Corporation, Osaka, Japan) was used to co-incubate with MG medium at 1, 10, and 100 μM, respectively. Bacterial growth was measured at OD_600_ for 24 h.

### Plant Materials

Plants used for *Pcal* virulence assays include cabbage (*Brassica oleracea* var. *capitate*) cv. Kinkei 201, oat (*Avena strigosa*) cv. Hayoat, Japanese radish (*Raphanus sativus* var. *longipinnatus*) cv. Natsutsukasa, Chinese cabbage (*Brassica rapa* var. *pekinensis*) cv. Akimeki, and broccoli (*Brassica oleracea* var. *italica*) cv. Midoribue. All plants were grown from seed at 23–25°C with a light intensity of 200 μE/m^2^/s and a 16 h light/8 h dark photoperiod. Plants were used for dip- and spray-inoculation assays around two weeks after germination, and for syringe-inoculation assays around 3 weeks after germination. For flood-inoculation, cabbage seeds were germinated and grown on 1/2 strength Murashige and Skoog (MS) medium (0.3% phytagel) with Gamborg vitamins (Sigma-Aldrich, St. Louis, MO, United States). Cabbage seedlings were incubated in a growth chamber at 24°C with a light intensity of 200 μE/m^2^/s and a 12 h light/12 h dark photoperiod, and used for the inoculation assays 2 weeks after germination.

### Bacterial Inoculation

To assay for disease on cabbage, oat, Japanese radish, broccoli, and Chinese cabbage plants, dip-inoculations were conducted by soaking seedlings in bacterial suspensions (5 × 10^7^ CFU/ml) containing 0.025% Silwet L-77 (OSI Specialities, Danbury, CT, United States). The seedlings were then incubated in growth chambers at 85–95% RH for the first 24 h, then at 80–85% RH for the rest of the experimental period. Disease symptoms were photographed at 5 days post-inoculation (dpi). To assess bacterial growth in all plants, the internal bacterial population was measured after dip-inoculation. Inoculated seedlings were collected, and two inoculated leaves were measured chronologically. The leaves were surface-sterilized with 10% H_2_O_2_ for 3 min. After washing three times with sterile distilled water, the leaves were homogenized in sterile distilled water, and diluted samples were plated onto solid KB agar medium. Two or three days after dilution sample plating, the bacterial colony forming units (CFUs) were counted and normalized as CFU per gram, using the total leaf weight. The bacterial populations at 0 dpi were estimated using leaves harvested 1-hour post-inoculation (hpi) without surface-sterilization. The bacterial populations were evaluated in at least three independent experiments.

For syringe-inoculation, cabbage and oat leaves were syringe-inoculated with bacterial suspensions (5 × 10^5^ or 5 × 10^7^ CFU/ml) with a 1-ml blunt syringe. L-tryptophan was co-inoculated with the *trpA* mutant at 100 μM, 1 mM, 10 mM, and 50 mM, respectively. The plants were then incubated at 70–80% RH for the rest of the experimental period. Leaves were removed and photographed at 5 dpi. To assess bacterial growth in cabbage, the internal bacterial population was measured after syringe-inoculation. Leaf disks were harvested using a 3.5 mm-diameter cork-borer from syringe-infiltrated leaf zones. To assess bacterial growth in oat, leaf pieces were cut from syringe-infiltrated leaf zones and the area (cm^2^) measured. The bacterial CFUs were counted and normalized as CFU per cm^2^ of leaf tissue. The bacterial populations were evaluated in at least three independent experiments.

### Epiphytic Bacterial Growth Assay

Flood-inoculation was conducted as described previously (Ishiga et al., 2011). Briefly, 50 ml of bacterial suspension (1 × 10^8^ CFU/ml) made in sterile distilled H_2_O containing 0.025% Silwet L-77 (OSI Specialities, Danbury, CT, United States) was dispensed onto a plate containing 2-week-old cabbage seedlings grown on 1/2 strength MS medium for uniform inoculation, then the plates were incubated for 2–3 min at room temperature. After the bacterial suspension was decanted from the plates, they were sealed with 3M Microspore 2.5 cm surgical tape (3M, St. Paul, MS, United States) and incubated at 24°C with a light intensity of 200 μE/m^2^/s and a 12 h light/12 h dark photoperiod. To assess epiphytic bacterial population on cabbage, the leaves were washed with washing buffer (50 mM PBS buffer) in an ultrasonic bath for 7 min, and then dilutions were plated to KB agar medium at 1 and 2 dpi. The bacterial CFUs were counted and normalized as CFU per gram, using the total leaf weight. The bacterial populations were evaluated in at least three independent experiments.

### Monitoring Bacterial Gene Expression in Culture

For expression profiles in culture conditions, *Pcal* was grown in KB medium for 24 h, then adjusted to an OD_600_ of 0.1 with fresh KB medium and grown for 3 h. The bacterial cells in the suspension were harvested by centrifugation at 12,000 rpm for 2 min, and cell pellets were resuspended with HS medium optimized for COR production (HSC; Palmer and Bender, 1993) and grown for 30 min. The bacterial cells in the suspension were harvested by centrifugation at 12,000 rpm for 2 min, and cell pellets were used for subsequent purification. Total RNA was extracted using Reliaprep (Promega, Madison, WI, United States) according to the manufacturer’s protocol. Two micrograms of total RNA were treated with gDNA Remover (TOYOBO, Osaka, Japan) to eliminate genomic DNA, and the DNase-treated RNA was reverse transcribed using the ReverTra Ace qPCR RT Master Mix (TOYOBO). The cDNA (1:10) was then used for RT-qPCR using the primers shown in Supplementary Table 2 with THUNDERBIRD SYBR qPCR Mix (TOYOBO) on a Thermal Cycler Dice Real Time System (Takara Bio). *Pcal* KB211 *outer membrane porin F* (*oprF*) and *recombinase A* (*recA*) were used to normalize gene expression. Gene expression of the *trpA* mutant was calculated as a relative value of WT expression. The reagent blank (no-template) controls were used to detect contamination. The expression profiles were evaluated in four independent samples.

### Monitoring Bacterial Gene Expression *in planta*

To analyze *Pcal* gene expression profiles during infection, we syringe-inoculated cabbage plants with *Pcal* at 5 × 10^7^ CFU/ml, and at 3 and 6 hrs the total RNAs including plant and bacterial RNAs were extracted from infected leaves and purified. Total RNA extraction and real-time quantitative RT-PCR (RT-qPCR) were done as described previously (Ishiga and Ichinose, 2016). Two micrograms of total RNA were treated with gDNA Remover (TOYOBO) to eliminate genomic DNA, and the DNase-treated RNA was reverse transcribed using the ReverTra Ace qPCR RT Master Mix (TOYOBO). The cDNA (1:10) was then used for RT-qPCR using the primers shown in Supplementary Table 2 with THUNDERBIRD SYBR qPCR Mix (TOYOBO) on a Thermal Cycler Dice Real Time System (Takara Bio). *Pcal* KB211 *outer membrane porin F* (*oprF*) and *recombinase A* (*recA*) were used to normalize gene expression. Gene expression of the *trpA* mutant was calculated as a relative value of WT 3 h expression. The reagent blank (no-template) controls were used to detect contamination. The expression profiles were evaluated in at least six independent samples.

## Results

### TrpA Is Involved in *Pcal* Pathogenicity

**FIGURE 1:**
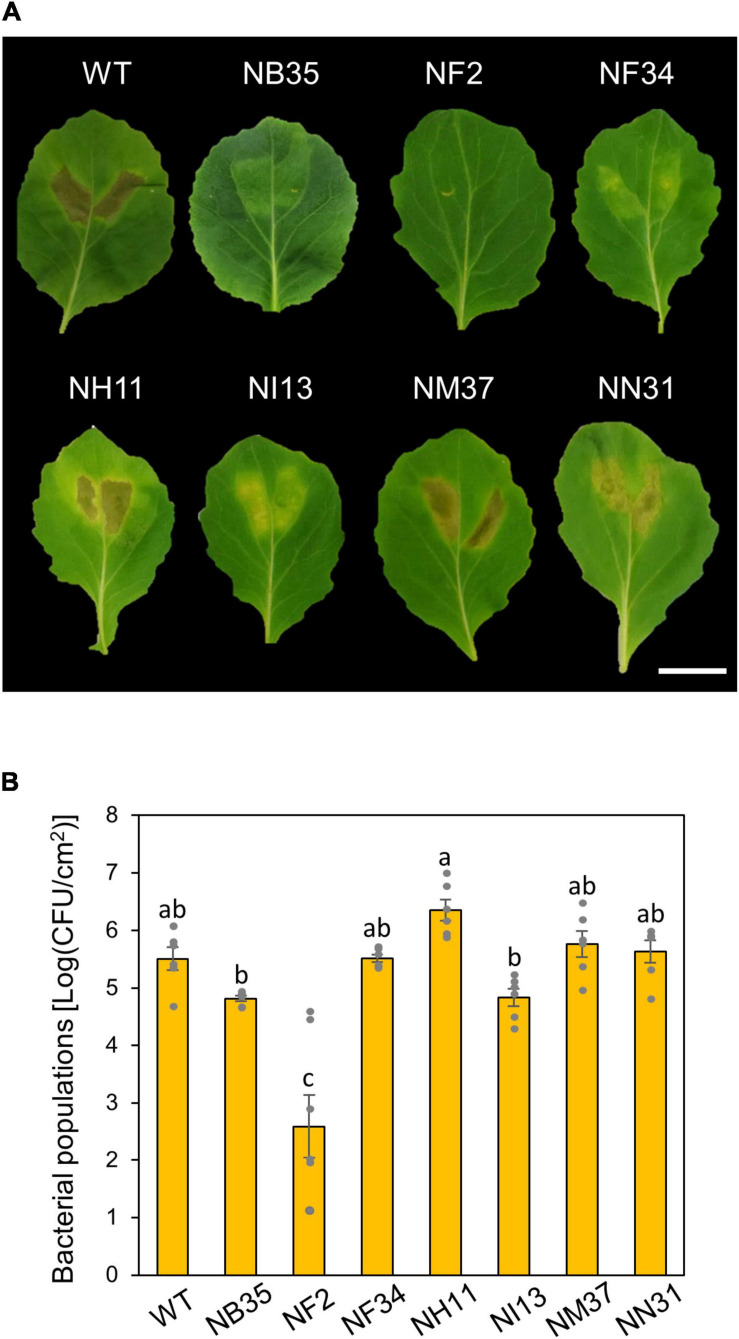
Disease phenotypes and bacterial populations of *Pseudomonas cannabina* pv. *alisalnesis* KB211 WT and amino acid mutants in cabbage after syringe-inoculation. Disease symptoms **(A)** and bacterial populations **(B)** on cabbage leaves syringe-inoculated with WT, a type three secretion system (T3SS) mutant (NB35) and six amino acid metabolism mutants (NF2, NF34, NH11, NI13, NM37, and NN31) described in Supplementary Table 1. Cabbage plants were syringe-inoculated with 5 × 10^5^ CFU/ml of inoculum. The bacterial concentrations in the plant leaves were evaluated at 5 dpi. The leaves were photographed at 5 dpi. Scale bar shows 2 cm. Vertical bars indicate the standard error for at least six independent experiments. Different letters indicate a significant difference among treatments based on a Tukey’s HSD test (*p* < 0.05).

To investigate functions of amino acid metabolism in *Pcal* virulence, we conducted inoculation assay using six amino acid metabolism mutants (Sakata et al., 2019). These six mutants (NF2, NF34, NH11, NI13, NM37, and NN31; see Supplementary Table 1) and a T3SS mutant (NB35) were syringe-inoculated into cabbage to investigate which mutants were most involved in *Pcal* virulence. NH11, NM37, and NN31 showed necrosis similar to WT. NB35, NF34, and NI13 only showed chlorosis. Only the NF2 mutant, which is mutated in the *trpA* (encoding tryptophan synthase alpha chain), showed no symptoms (Figure 1A). Moreover, *trpA* mutant bacterial populations were severely reduced among all mutants, including the T3SS mutant (Figure 1B).

### TrpA Contributes to *Pcal* Multiplication in the Apoplast to Cause Disease

**FIGURE 2:**
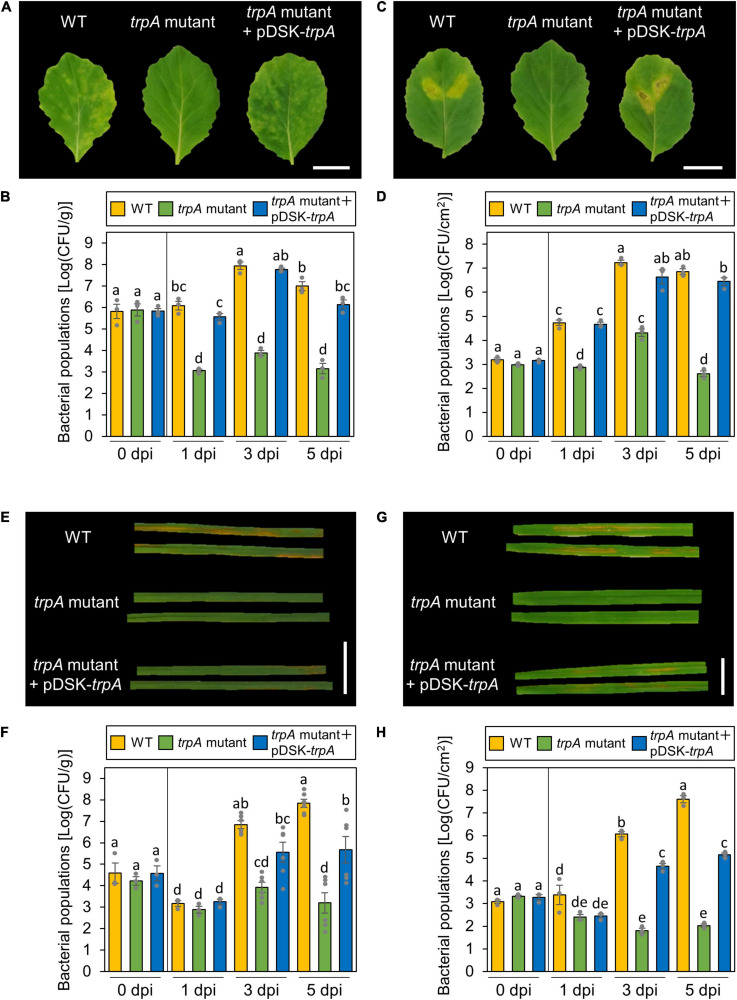
Disease phenotypes and bacterial populations of *Pseudomonas cannabina* pv. *alisalnesis* KB211 WT and the *trpA* mutant in cabbage and oat after dip- and syringe-inoculation. Disease symptoms **(A)** and bacterial populations **(B)** in cabbage dip-inoculated with WT, the *trpA* mutant, and the *trpA* mutant complemented with pDSK-*trpA*. Disease symptoms **(C)** and bacterial populations **(D)** in cabbage syringe-inoculated with WT, the *trpA* mutant, and the *trpA* mutant complemented with pDSK-*trpA.* Disease symptoms **(E)** and bacterial populations **(F)** in oat dip-inoculated with WT, the *trpA* mutant, and the *trpA* mutant complemented with pDSK-*trpA*. Disease symptoms **(G)** and bacterial populations **(H)** in oat syringe-inoculated with WT, the *trpA* mutant, and the *trpA* mutant complemented with pDSK-*trpA*. Cabbage and oat were dip-inoculated with 5 × 10^7^ CFU/ml of inoculum containing 0.025% SilwetL-77 and syringe-inoculated with 5 × 10^5^ CFU/ml of inoculum, respectively. Bacterial concentrations in the plant leaves were evaluated at 0, 1, 3, and 5 dpi. The leaves were photographed 5 dpi. Vertical bars indicate the standard error for at least three independent experiments. Different letters indicate a significant difference among treatments based on a Tukey’s HSD test (*p* < 0.05). Scale bar shows 2 cm.

*trpA* is apparently dispensable for *Pcal* growth in rich LB medium, since no growth difference was observed between the WT and the *trpA* mutant (Supplementary Figure 1). Cabbage inoculated with WT showed severe chlorosis, but the *trpA* mutant induced no symptoms (Figure 2A). Although bacterial populations of the WT and the *trpA* mutant complemented with pDSK-*trpA* reached around 1 × 10^8^ CFU/ml when dip-inoculated onto cabbage, *trpA* mutant populations were 10^4^ times less (Figure 2B). Moreover, the *trpA* mutant multiplication defect was observed within 1 dpi (Figure 2B). To further investigate whether the *trpA* mutant is impaired in apoplastic growth and pathogenicity, syringe-infiltration was used to bypass stomatal defense. When infiltrated directly into the cabbage apoplast, *trpA* mutant disease development and bacterial populations were decreased compared to the WT (Figures 2C,D).

Importantly, *Pcal* is pathogenic on monocot plants, such as oat, as well as cruciferous species (Ishiyama et al., 2013). Oat plants inoculated with the *trpA* mutant also showed reduced symptoms as well as bacterial populations for both dip- and syringe-inoculation methods (Figures 2E–H).

### TrpA Contributes to Multiplication on the Leaf Surface and Disease

**FIGURE 3:**
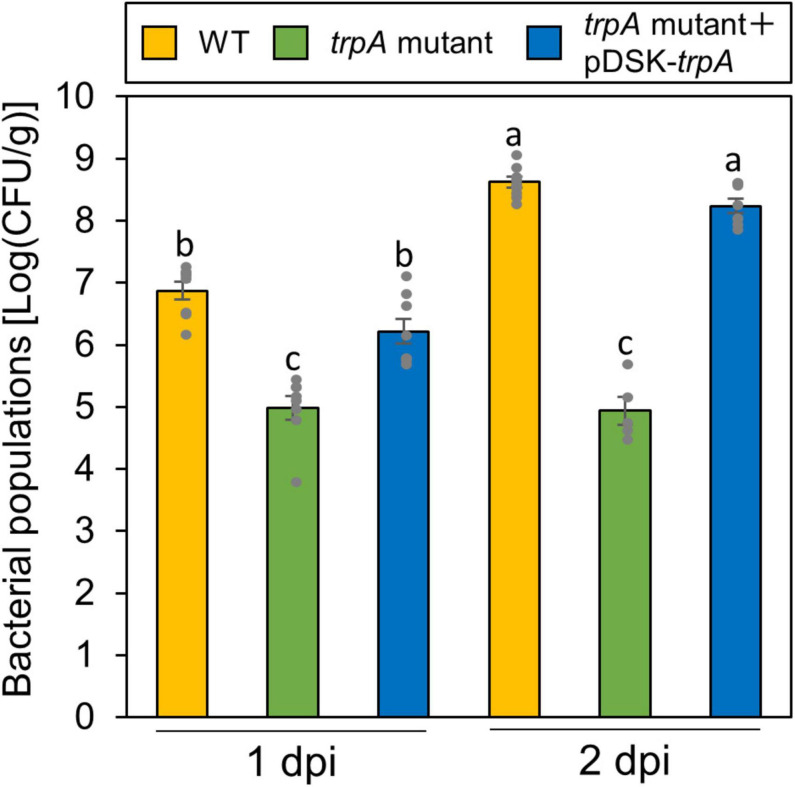
Epiphytic bacterial populations of *Pseudomonas cannabina* pv. *alisalnesis* KB211 WT and the *trpA* mutant on cabbage after flood-inoculation. Cabbage seedlings were flood-inoculated with 1 × 10^8^ CFU/ml of WT, the *trpA* mutant, and the *trpA* mutant complemented with pDSK-*trpA* containing 0.025% SilwetL-77. Epiphytic bacterial populations were determined by washing the leaves with washing buffer (50 mM PBS buffer) in an ultrasonic bath for 7 min, and then by plating dilutions to KB agar medium. Bacterial concentrations on the leaf surface were evaluated at 1 and 2 dpi. Vertical bars indicate the standard error for at least five biological replicates. Different letters indicate a significant difference among treatments based on a Tukey’s HSD test (*p* < 0.05).

Globally, the microbiota on plant leaf surfaces is estimated to around 10^26^ bacterial cells (Lindow and Brandl, 2003). The plant surface is generally considered to be suboptimal for microbes (Lindow and Brandl, 2003). Because the *trpA* mutant is auxotrophic, and does not grow on minimal medium (MG medium) (Sakata et al., 2019), the *trpA* mutant is likely to be affected by other plant microbiomes, which compete for limited nutrients. Thus, to rule out the possibility that the *trpA* mutant showed reduced virulence because of a microbiome effect, we conducted flood-inoculation onto cabbage, which can assay the plant-bacterial interaction with a sterility test (Ishiga et al., 2011), and investigated epiphytic bacterial populations. The *trpA* mutant showed reduced population via flood-inoculation (Figure 3).

### Tryptophan Is an Essential Growth Resource

**FIGURE 4:**
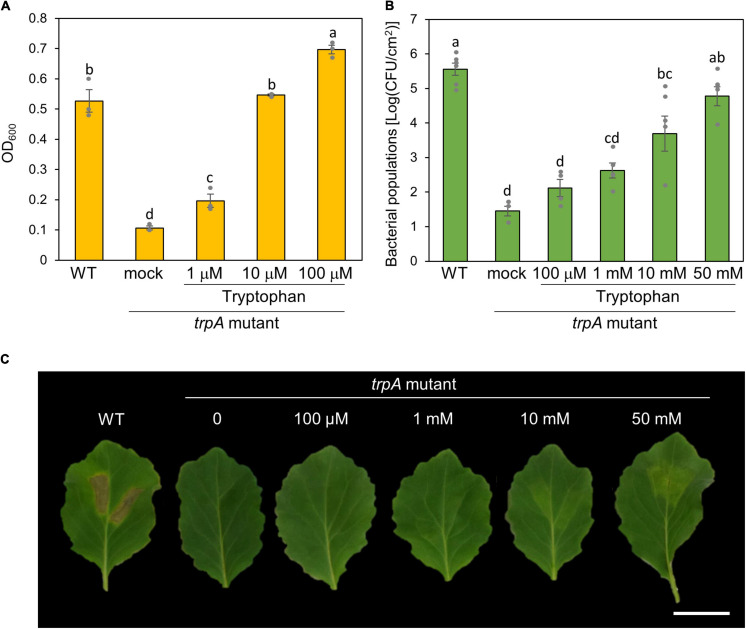
Effect of exogenous tryptophan application on *trpA* mutant growth. **(A)** Growth of *Pseudomonas cannabina* pv. *alisalnesis* KB211 WT and the *trpA* mutant in MG medium. WT and the *trpA* mutant were adjusted to an OD_600_ of 0.1 in MG medium. Additionally, the *trpA* mutant was co-incubated with tryptophan; 1, 10, and 100 μM, respectively. Then, cultures were incubated with shaking at 28°C. Bacterial populations were quantified at 24 h. **(B)** Bacterial populations in cabbage syringe-inoculated with WT and the *trpA* mutant. Cabbage plants were syringe inoculated with 5 × 10^5^ CFU/ml of WT and the *trpA* mutant. The *trpA* mutant was co-inoculated with tryptophan; 100 μM, 1 mM, 10 mM, and 50 mM, respectively. Bacterial concentrations in the plant leaves were evaluated at 5 dpi. **(C)** Disease symptoms on cabbage co-inoculated with tryptophan. Cabbage plants were syringe-inoculated with 5 × 10^5^ CFU/ml of WT and the *trpA* mutant. The *trpA* mutant was co-inoculated with 100 μM, 1, 10, and 50 mM tryptophan, respectively. The leaves were photographed at 5 dpi. Scale bar shows 2 cm.

To investigate whether exogenous tryptophan application restores this virulence impairment, we first measured bacterial growth in MG medium with tryptophan. The *trpA* mutant was growth deficient in MG medium (Figure 4A). Co-incubation with only 10 μM tryptophan restored *trpA* mutant bacterial growth to the WT level *in vitro* (Figure 4A).

We further tested whether exogenous tryptophan application restores *trpA* mutant virulence on cabbage. Co-inoculation of the *trpA* mutant with tryptophan restored virulence in a dose dependent manner (Figures 4B,C). Although only 10 μM tryptophan restored *trpA* mutant bacterial growth to the WT level *in vitro*, at least 50 mM tryptophan was required for the *trpA* mutant to multiply to a similar level as WT in cabbage by 5 dpi (Figure 4B). Although bacterial multiplications were recovered, 50 mM exogenous application could not fully recover disease symptoms (Figure 4C and Supplementary Figure 2A). To further investigate symptom development and bacterial populations, we conducted inoculation with or without tryptophan (50 mM) in a time-dependent manner. Exogenous tryptophan application partially recovered *trpA* mutant multiplication at 1 and 3 dpi (Supplementary Figure 2B).

### *trpA* Mutant Virulence Genes Show Reduced Expression During Infection

Ten μM exogenous tryptophan application recovered bacterial growth *in vitro*, while at least 50 mM of tryptophan was required for growth recovery *in planta* (Figures 4A,B). Therefore, we assumed that tryptophan is not only a nutrient but also involved in regulating bacterial virulence gene expression. We firstly examined *Pcal* gene expression profiles in culture medium. As we expected, T3Es genes (including *avrPto*, *hopM1*, and *avrE1*) and COR biosynthesis related genes (including *cmaA*, *cfl*, and *corR*) showed reduced expression in the *trpA* mutant compared to WT (Supplementary Figures 3A–F). *trpB* showed significantly greater expression in the *trpA* mutant (Supplementary Figure 3G).

**FIGURE 5:**
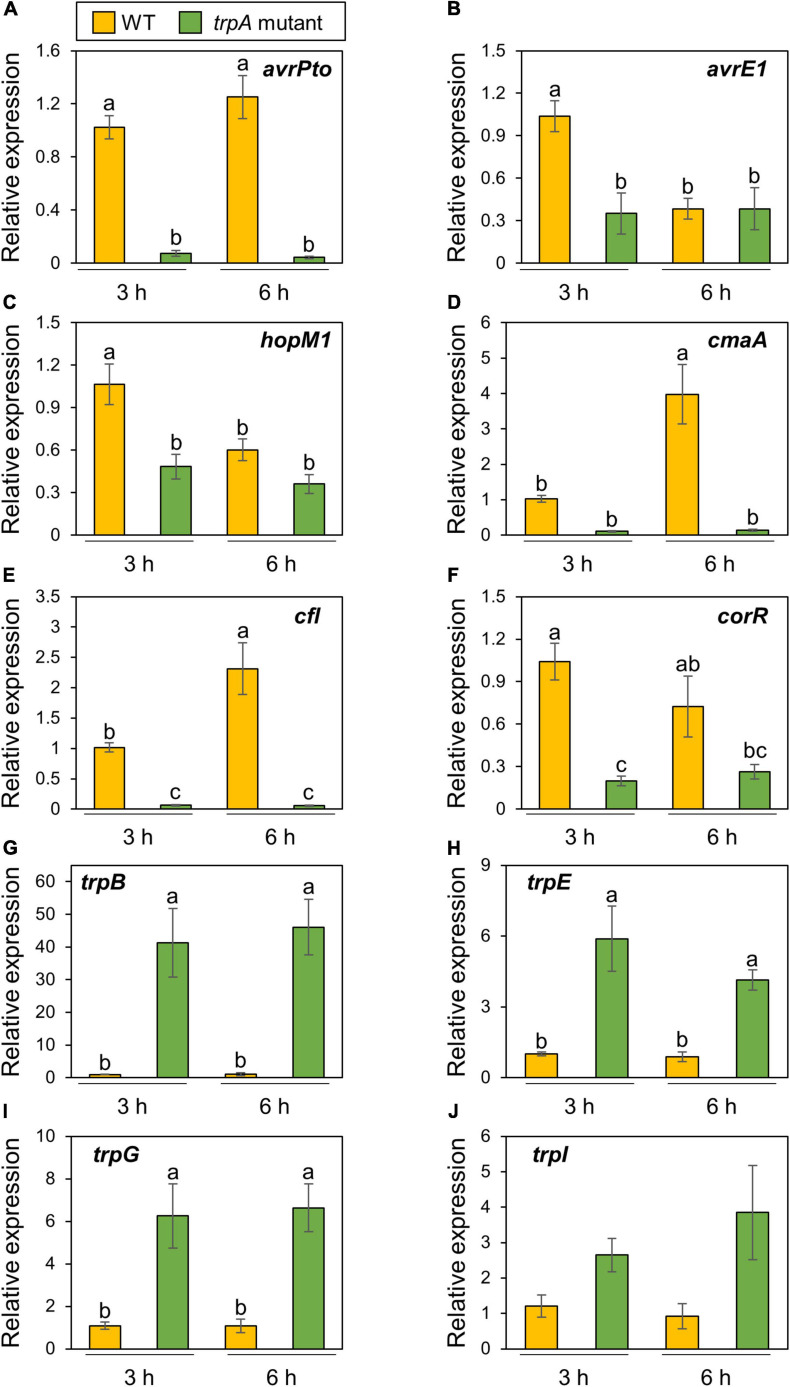
Expression profiles of bacterial virulence genes and tryptophan biosynthesis genes during *Pseudomonas cannabina* pv. *alisalnesis* KB211 WT and *trpA* mutant infection. Cabbage plants were syringe-inoculated with 5 × 10^7^ CFU/ml of WT and the *trpA* mutant and total RNAs were collected 3 and 6 h after inoculation. Expression profiles of type three effector (T3Es) genes [including *avrPto* **(A)**, *avrE1* **(B)**, and *hopM1* **(C)**] and COR biosynthesis related genes [including *cmaA* **(D)**, *cfl* **(E)**, and *corR* **(F)**] were investigated. Additionally, expression profiles of tryptophan biosynthesis related genes [including *trpB* **(G)**, *trpE* **(H)**, *trpG* **(I)**, and *trpI* **(J)**], were also investigated. Total RNA was extracted for use in real-time quantitative reverse transcription-polymerase chain reaction (RT-qPCR) with gene-specific primer sets (Supplementary Table 2). Expression was normalized using *oprE* and *recA*. Vertical bars indicate the standard error for at least six biological replicates. Different letters indicate a significant difference among treatments based on a Tukey’s HSD test (*p* < 0.05).

Then, we next investigated these gene expression profiles during infection. The *trpA* mutant showed significant bacterial population reduction compared to the WT within 1 dpi. To determine the time point at which the WT and *trpA* mutant had the same bacterial populations, we syringe-inoculated these into cabbage at 5 × 10^7^ CFU/ml and examined earlier time points including 3, 6, and 9 hpi. *trpA* mutant bacterial populations were decreased compared to WT at 6 hpi, and this defect was greater at 9 hpi (Supplementary Figure 4). Therefore, we conducted syringe-inoculation into cabbage with these strains at 5 × 10^7^ CFU/ml and extracted the total RNAs at 3 and 6 hpi. T3Es genes (Figures 5A–C) and COR biosynthesis related genes (Figures 5D–F) exhibited reduced expression in the *trpA* mutant compared to WT. We also investigated tryptophan biosynthesis gene expression profiles. *trpB*, *trpE*, *trpG*, and *trpI* showed greater expression in the *trpA* mutant than in the WT (Figures 5G–J).

### TrpA Contributes to Disease on Multiple Host Plants

**FIGURE 6:**
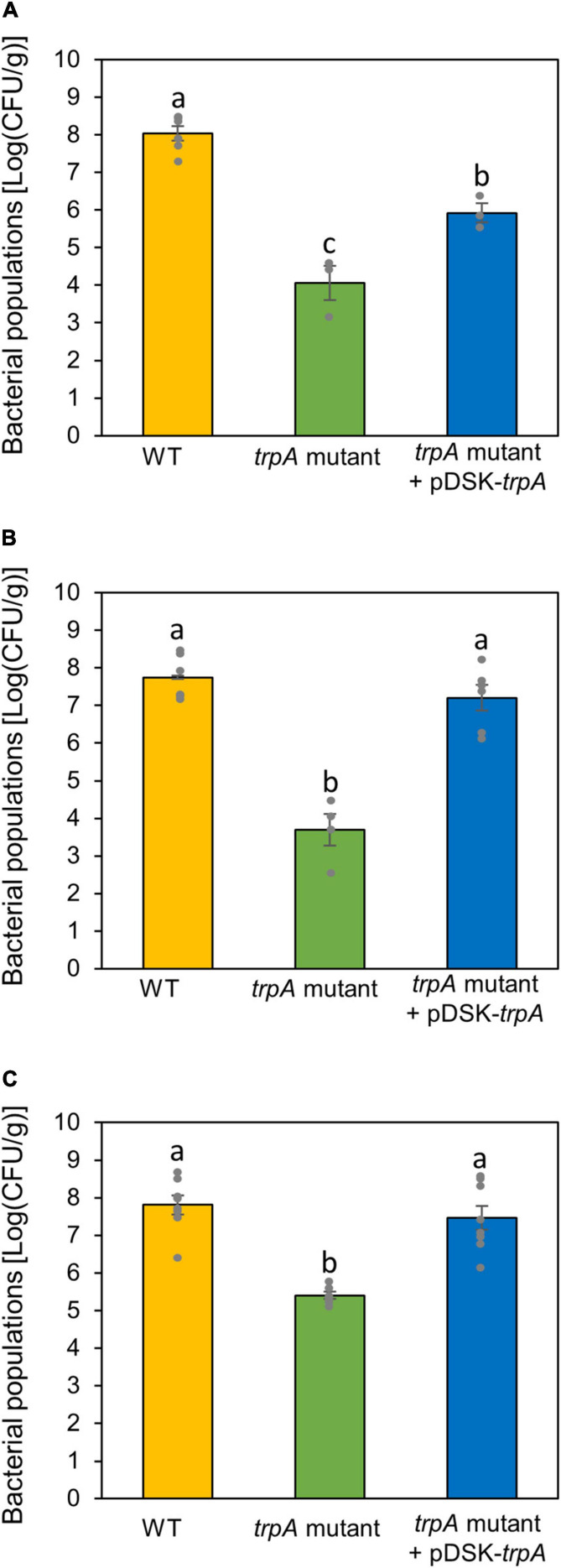
Bacterial populations of *Pseudomonas cannabina* pv. *alisalnesis* KB211 WT and the *trpA* mutant in Japanese radish **(A)**, broccoli **(B)**, and Chinese cabbage **(C)**. All plants were spray inoculated with 5 × 10^7^ CFU/ml of inoculum containing 0.025% Silwet L-77. Bacterial concentrations in the plant leaves were evaluated at 5 dpi. Vertical bars indicate the standard error for at least three biological replicates. Different letters indicate a significant difference among treatments based on a Tukey’s HSD test (*p* < 0.05).

Since the *trpA* mutant showed no pathogenicity on both cabbage and oat, we hypothesized that TrpA was required for pathogenesis on multiple host plants. We therefore inoculated the WT and *trpA* mutant onto several host plants, including Japanese radish, broccoli, and Chinese cabbage, and measured bacterial populations. The *trpA* mutant showed reduced symptom development and bacterial multiplication in all host plants (Figure 6 and Supplementary Figure 5).

## Discussion

Plant pathogens deploy multiple virulence factors for successful infection. This study was performed to understand the role of TrpA in *Pcal* virulence. Overall, this study showed that TrpA contributes to *Pcal* pathogenesis and is important for successful infection in multiple host plants. Importantly, *trpA* disruption leads to downregulation of bacterial virulence genes, including T3Es and COR, and to upregulation of tryptophan biosynthesis related genes, demonstrating the importance of tryptophan biosynthesis in bacterial pathogenesis.

Among six amino acid metabolism mutants we identified (Sakata et al., 2019), the *trpA* mutant showed no pathogenesis (Figure 1). These results indicate that tryptophan biosynthesis is critical for *Pcal* virulence. Schreiber et al. (2012) conducted a high-throughput forward genetic screen and demonstrated the nutritional requirements of *Psm* ES4326 (*Pcal* ES4326) in *Arabidopsis*. Amino acid auxotrophs showed dramatically reduced bacterial populations following spray inoculation (Schreiber et al., 2012), suggesting that free amino acids are limited on leaf surfaces and are important for growth. Together, these results underscore the importance of amino acid metabolism in plant bacterial pathogen virulence.

Our data showed that TrpA contributes to multiplication on the leaf surface and in the apoplast, causing disease (Figures 2, 3). Helmann et al. (2019) conducted RB-TnSeq to define the fitness contributions of *Pss* B728a genes. Consistent with our results, they demonstrated that genes within the tryptophan biosynthetic pathway had the greatest effect on fitness both on the leaf surface and in the apoplast (Helmann et al., 2019). Generally, the plant surface is considered suboptimal for microbes, which provides limited nutrient resources to bacterial colonists (Lindow and Brandl, 2003). Morgan and Tukey (1964) demonstrated that tryptophan was not detected in several plant species among 20 amino acids detected in plant leachates, suggesting a pressing need for its synthesis by bacterial colonists. Moreover, the ability of *Pcal* to synthesize tryptophan strongly influences bacterial proliferation in multiple host plants (Figure 6). Together, tryptophan biosynthesis is an essential process for successful *Pcal* infection on multiple host plants.

Bacterial multiplication of the *trpA* mutant was restored to WT levels with only 10 μM tryptophan *in vitro* (Figure 4A). Exogenous tryptophan application rescued the *trpA* mutant bacterial multiplication defect *in planta*, but at least 50 mM tryptophan was required (Figure 4B). Therefore, we assumed that the reduced virulence of the *trpA* mutant is involved in plant-bacterial interactions, as well as lack of nutrition. Transcripts of T3Es and COR related genes were reduced during *trpA* mutant infection compared to WT (Figure 5). Conversely, tryptophan biosynthesis related genes showed greater expression during *trpA* mutant infection than WT (Figure 5). The tryptophan biosynthesis pathway and genes have been proposed to be highly conserved in proteobacteria (Bae et al., 1989; Essar et al., 1990; Gussin, 2004). In *P. syringae*, the *trpBA* operon is regulated by TrpI, a LysR-type transcriptional activator whose gene is transcribed divergently (Auerbach et al., 1993). When tryptophan concentration is low, indoleglycerol phosphate (InGP) accumulates and TrpI assumes its active conformation, where it is able to bind at two operator sites in the *trpI*-*trpBA* intergenic region (Chang and Crawford, 1990; Merino et al., 2008). Our results demonstrated that these genes showed greater expression in the *trpA* mutant than in the WT (Figure 5). Taken together, our results suggest that *trpA* mutation directly affects tryptophan biosynthesis genes, and indirectly affects virulence factor related genes. One possible explanation how mutation in *trpA* affects T3Es and COR related genes is trade-off between nutrition acquisition and virulence. Several studies have demonstrated that nutrient assimilation during host infection is critical for pathogenesis (Brown et al., 2008; Eisenreich et al., 2010; Barbier et al., 2011; Schoen et al., 2014). In the plant pathogen *Ralstonia solanacearum*, exopolysaccharide (EPS) (which are critical for disease symptom production) biosynthesis and secretion represent a significant energetic cost for the pathogen, resulting in reduced bacterial growth (Peyraud et al., 2016). Furthermore, the animal pathogen *Salmonella* also balances the trade-off between fast growth and T3SS production (Ackermann et al., 2008; Diard et al., 2013). Taken together, the trade-off between growth and virulence should be considered in plant and bacterial pathogen interactions. Although further investigation will be necessary to understand how mutation in *trpA* affect the bacterial virulence factors, trade-off between virulence factor production and bacterial proliferation with nutrients such as tryptophan might be present in *Pcal* infection processes.

In conclusion, these data strongly suggest that TrpA contributes to bacterial multiplication on the leaf surface and in the apoplast, and contributes to disease in multiple host plants. Furthermore, *trpA* mutation leads to downregulation of virulence genes related to T3Es and COR. Since most amino acids are apparently present at relatively low apoplastic concentrations (O’Leary et al., 2016), it is expected that lack of amino acid metabolites function causes reduced virulence in addition to growth defects. Our findings suggest that amino acid metabolites can be targeted for developing new disease control strategies.

## Data Availability Statement

The datasets presented in this study can be found in online repositories. The names of the repository/repositories and accession number(s) can be found below: https://figshare.com/s/753ec4995e4086b047da.

## Author Contributions

NS and YI designed the experiments and wrote the manuscript. All authors performed the experiments.

## Conflict of Interest

The authors declare that the research was conducted in the absence of any commercial or financial relationships that could be construed as a potential conflict of interest.
