# Supplementary material for: Pseudmonas cannabina pv. alisalensis TrpA Is Required for Virulence in Multiple Host Plants
Source: Front Microbiol. 2021 Apr 20;12:659734. doi: 10.3389/fmicb.2021.659734 (PMC8093880; doi:10.3389/fmicb.2021.659734)
Supplement: Supplementary file 1 [file Data_Sheet_1.pdf]

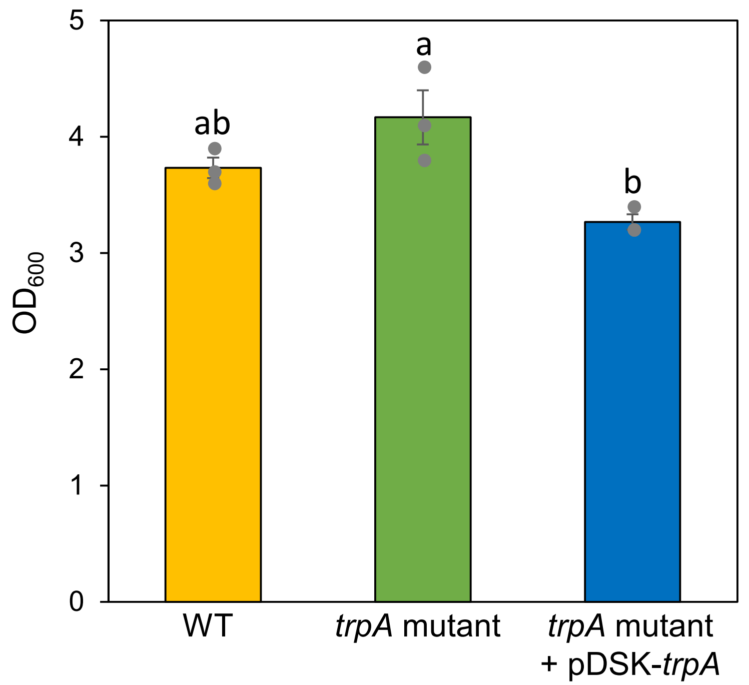

Supplementary Figure 1. *Pseudomonas cannabina* pv. *alisalnesis* KB211 WT and *trpA* mutant growth in LB medium. All strains were adjusted to an OD of 0.01 in LB medium and incubated with shaking at 28°C. Bacterial growth was quantified at 24 hrs. Vertical bars indicate the standard error for three biological replicates. Different letters indicate a significant difference among treatments based on a Tukey's HSD test ($p < 0.05$)..

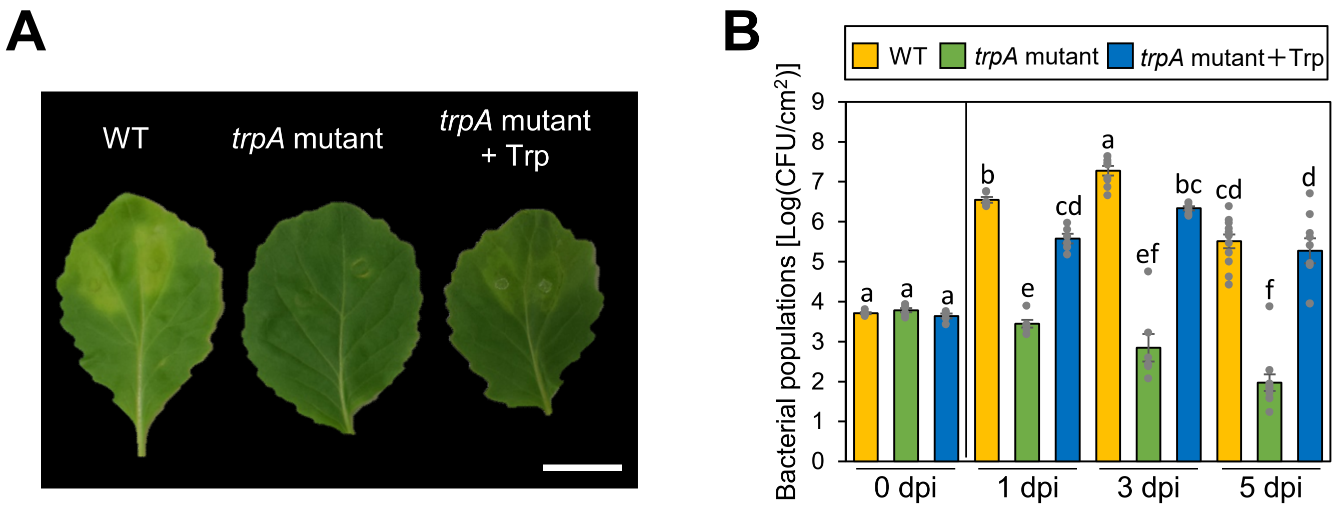

Supplementary Figure 2. Disease symptoms (A) and bacterial populations (B) of WT and the *trpA* mutant with or without tryptophan. Cabbage plants were syringe-inoculated with 5 x 10^5 CFU/ml of WT and the *trpA* mutant. The *trpA* mutant was co-inoculated with tryptophan (50 mM) in cabbage. Bacterial populations in the plant leaves were evaluated at 0, 1, 3, and 5 dpi. The leaves were photographed at 5 dpi. Scale bar shows 2 cm. Vertical bars indicate the standard error for at least six independent experiments. Different letters indicate a significant difference among treatments based on a Tukey's HSD test ($p < 0.05$).

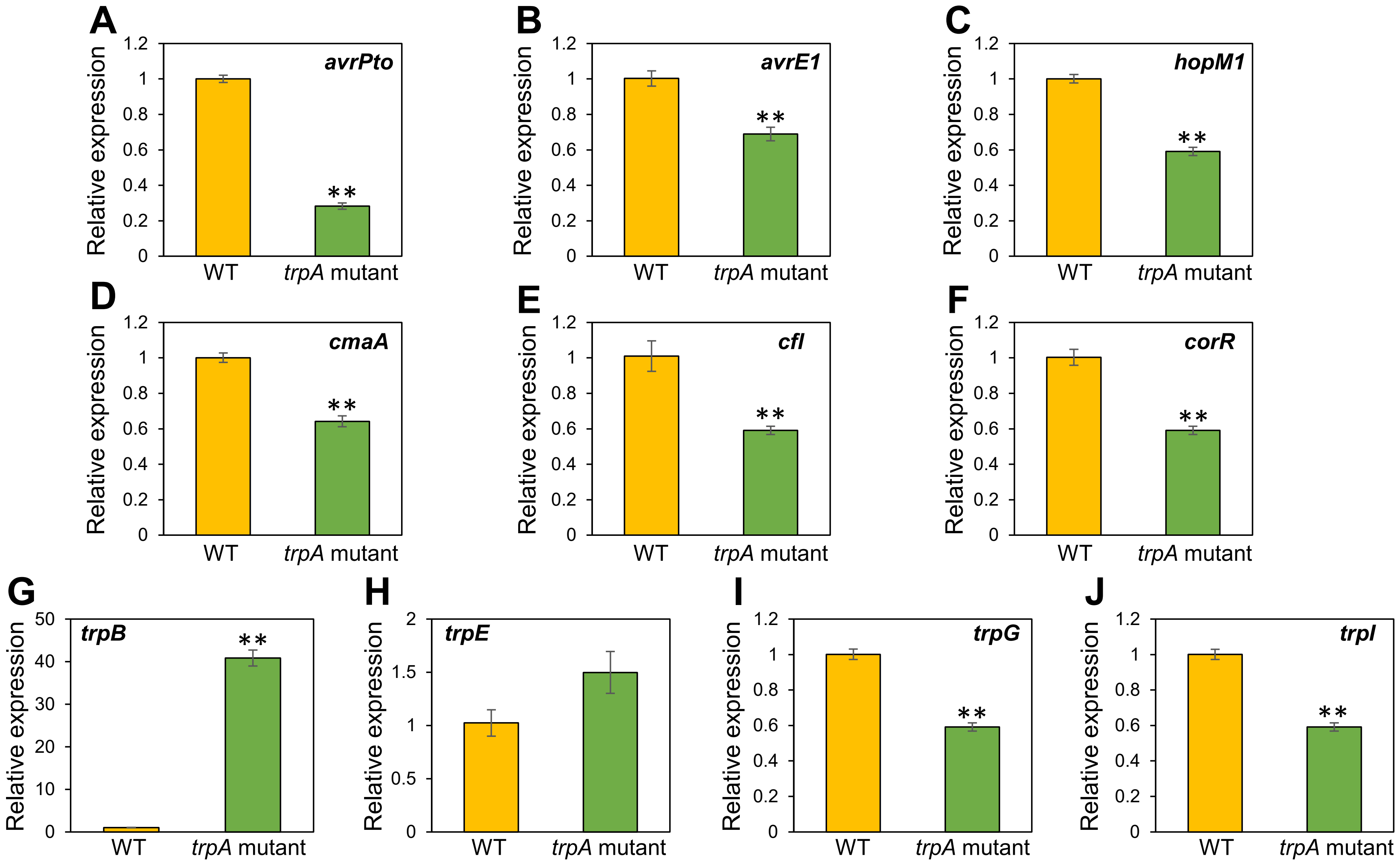

Supplementary Figure 3. Expression profiles of bacterial virulence genes and tryptophan biosynthesis genes during *Pseudomonas cannabina* pv. *alisalnesis* KB211 WT and *trpA* mutant *in vitro* growth in HSC medium. Expression profiles of type three effectors (T3Es) related genes (including *avrPto* (A), *avrE1* (B), and *hopM1* (C)) and COR biosynthesis related genes (including *cmaA* (D), *cfl* (E), and *corR* (F)) were investigated. Additionally, expression profiles of tryptophan biosynthesis related genes (including *trpB* (G), *trpE* (H), *trpG* (I), and *trpI* (J)), were also investigated. Total RNA was extracted for use in real-time quantitative reverse transcription-polymerase chain reaction (RT-qPCR) with gene-specific primer sets shown in Supplementary Table 2. Expression was normalized using *oprE* and *recA*. Vertical bars indicate the standard error for at least six biological replicates. Asterisks indicate a significant difference from the WT and the *trpA* mutant in a *t* test (** $p < 0.01$).

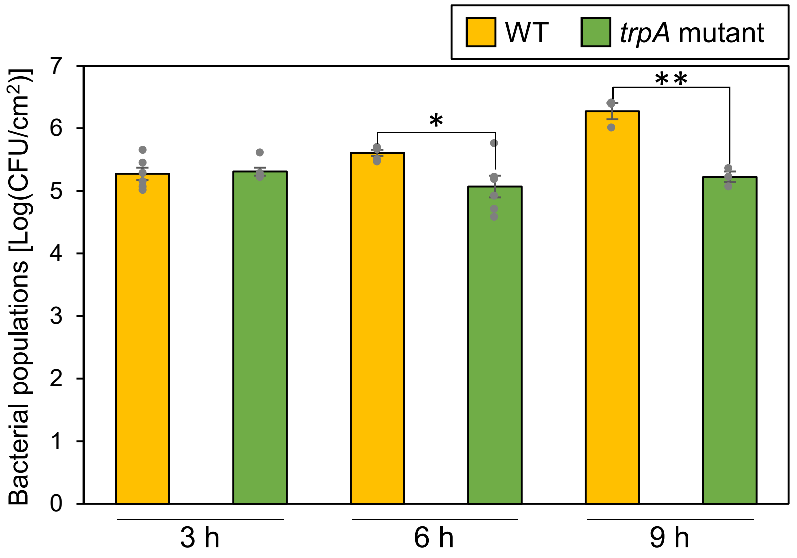

Supplementary Figure 4. Bacterial populations of *Pseudomonas cannabina* pv. *alisalnesis* KB211 WT and the *trpA* mutant in cabbage after syringe inoculation. Cabbage plants were syringe inoculated with 5 x 10^7 CFU/ml of WT and the *trpA* mutant. Bacterial concentrations in the plant leaves were evaluated at 3, 6, and 9 hpi. Vertical bars indicate the standard error for at least three independent experiments. Asterisks indicate a significant difference from the WT and *trpA* mutant in a *t* test (* $p < 0.05$, ** $p < 0.01$).

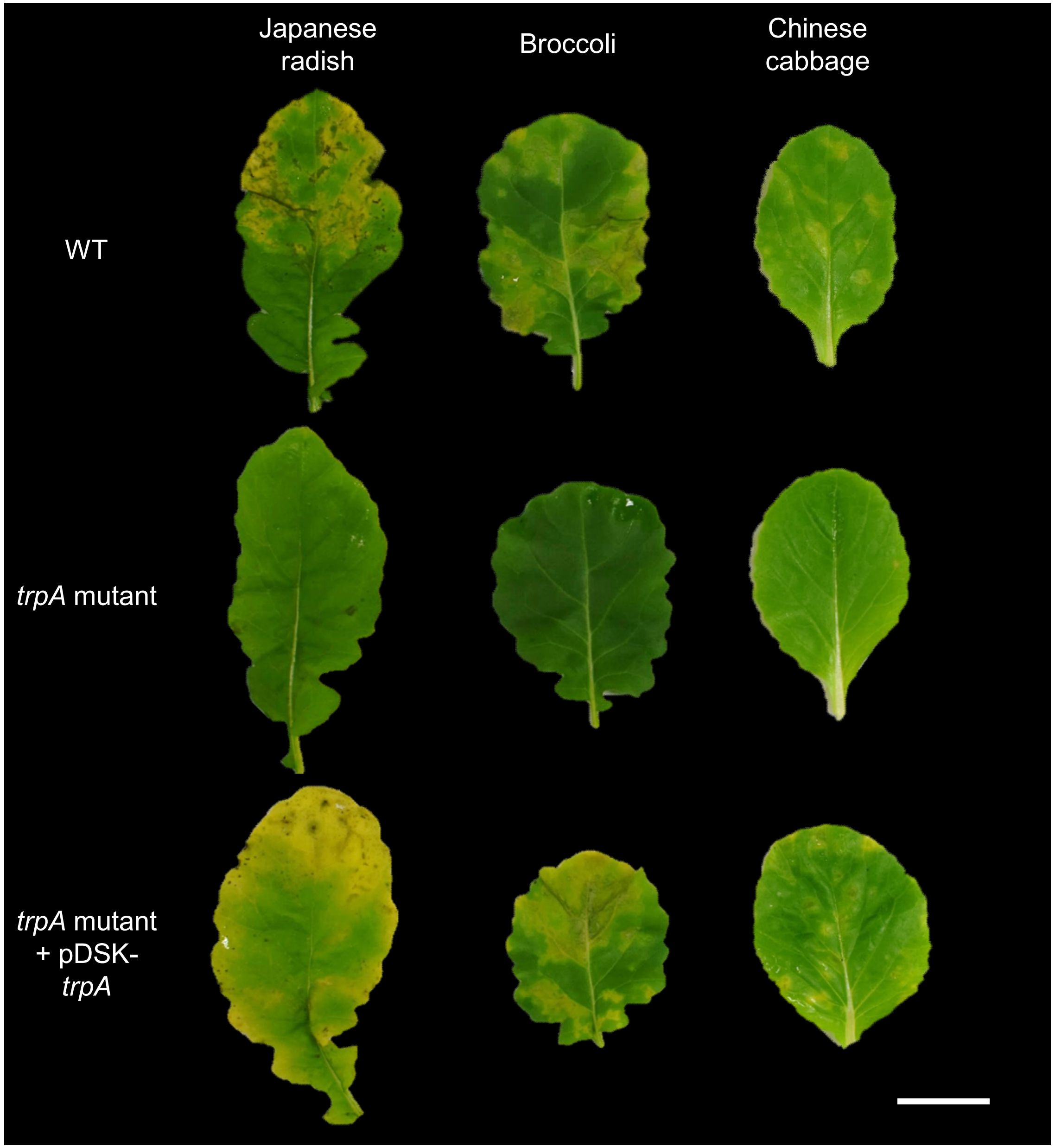

Supplementary Figure 5. Disease symptoms on Japanese radish, broccoli, and Chinese cabbage after spray-inoculation with *Pseudomonas cannabina* pv. *alisalnesis* KB211 WT and the *trpA* mutant. All plants were spray-inoculated with 5 x 10^7 CFU/ml of inoculum containing 0.025% SilwetL-77. The leaves were photographed at 5 dpi. Scale bar shows 2 cm.
