# Supplementary material for: Pseudmonas cannabina pv. alisalensis TrpA Is Required for Virulence in Multiple Host Plants
Source: Front Microbiol. 2021 Apr 20;12:659734. doi: 10.3389/fmicb.2021.659734 (PMC8093880; doi:10.3389/fmicb.2021.659734)
Supplement: Supplementary file 3 [file Table_1.PDF]

Supplementary Table S1. Bacterial strains and plasmids used in this study

Bacterial strain or plasmid	Relevant characteristics	Reference or source
P. cannabina pv. *alisalensis*		
Isolate KB211	Wild type, Rif ^{r}	Nagano vegetable and ornamental crops experiment station
P. cannanbina pv. *alisalensis* Tn*5* mutants		
NB35	Tn*5* mutant containing transposon in gene encoding type III secretion protein HrcQb, Rif ^{r}, Km ^{r}, Cm ^{r}	Sakata et al. (2019)
NF2 (*trpA* mutant)	Tn*5* mutant containing transposon in gene encoding tryptophan synthase subunit alpha TrpA, Rif ^{r}, Km ^{r}, Cm ^{r}	Sakata et al. (2019)
trpA mutant + pDSK-*trpA*	*trpA* mutant complemented with pDSK-*trpA*, Rif ^{r}, Km ^{r}, Cm ^{r}, Cm ^{r}	This study
NF34	Tn*5* mutant containing transposon in gene encoding 3-phosphoglycerate dehydrogenase, Rif ^{r}, Km ^{r}, Cm ^{r}	Sakata et al. (2019)
NH11	Tn*5* mutant containing transposon in gene encoding N-ethylammeline chlorohydrolase, Rif ^{r}, Km ^{r}, Cm ^{r}	Sakata et al. (2019)
NI13	Tn*5* mutant containing transposon in gene encoding N-acetyl-gamma-glutamyl-phosphate reductase, Rif ^{r}, Km ^{r}, Cm ^{r}	Sakata et al. (2019)
NM37	Tn*5* mutant containing transposon in gene encoding taurine catabolism dioxygenase TauD, Rif ^{r}, Km ^{r}, Cm ^{r}	Sakata et al. (2019)
NN31	Tn*5* mutant containing transposon in gene encoding D-amino acid dehydrogenase small subunit, Rif ^{r}, Km ^{r}, Cm ^{r}	Sakata et al. (2019)
Plasmid		
pBSLC1	Transposon vector constructed by ligation pf pBSL118 and pHSG396 at EcoRI site, Amp ^{r}, Km ^{r}, Cm ^{r}	Sawada et al. (2018)
pDSK-*trpA*	The vector containing constitutive *psbA* promoter and *trpA* gene inserted into pDSK519, Gen ^{r}	This study

Amp ^{r} ampicillin resistance, *Cm* ^{r} chloramphenicol resistance, *Gen* ^{r} genramicin resistance, *Km* ^{r} kanamycin resistance, *Rif* ^{r} rifampicin resistance

Supplementary Table S2. Primer sets used for *Pcal* gene expression analysis in this study

Gene	Primer name	Primer sequence	Reference
avrPto	avrPto-FW	CATCGATACCTGACCAACGATAC	This study
avrPto	avrPto-RV	CTCAGTCAGCTTGGTGCTAAT	This study
avrE1	avrE1-FW	GACAATCAGGGCAGGCTTTA	This study
avrE1	avrE1-RV	TGACCTGCCAAGGTGTAATG	This study
hopM1	hopM1-FW	GGTGCCGATGAAGGCTATT	This study
hopM1	hopM1-RV	TTTCGACGGACGCTTTGT	This study
cmaA	cmaA-FW	AAAGCCTACCGCCGATTT	Sakata et al., 2021
cmaA	cmaA-RV	CGTCTGGAGCTGTTGATAAGT	Sakata et al., 2021
cfl	cfl-FW	GAACTGGTGGCGTTGTACTAT	Sakata et al., 2021
cfl	cfl-RV	GTGGAGCAGATGCTCAATTTC	Sakata et al., 2021
corR	corR-FW	GGATCGAACGCTGGCAGATA	Sakata et al., 2021
corR	corR-RV	GTCCTGCTCATGAGTCGCTT	Sakata et al., 2021
trpB	trpB-FW	CAACGTCGATGACACCTTCT	This study
trpB	trpB-RV	TCTCCTGCATCTGCTCTTTG	This study
trpE	trpE-FW	TTCGTACAGCGGTGATCAAG	This study
trpE	trpE-RV	GCGTTTGTTGAGGGTTTCTTC	This study
trpG	trpG-FW	CATCCGCAATGACGAACTGA	This study
trpG	trpG-RV	GATTGATCACTTCCAGCGAGAC	This study
trpI	trpI-FW	TGGCCTGAAACTCACAGATG	This study
trpI	trpI-RV	TGCTCAGTTCGACACACAC	This study
oprF	oprF-FW	GGCTTGGCCATTGGTACTAT	Sakata et al., 2021
oprF	oprF-RV	GCGCTGTCGTAATACTCTTTCT	Sakata et al., 2021
recA	recA-FW	TCTCTACGGCAAGGGTATCT	Sakata et al., 2021
recA	recA-RV	GCTTTACCCTGACCGATCTT	Sakata et al., 2021
